# Education of parents in Pavlik harness application for developmental dysplasia of the hip using a validated simulated learning module

**DOI:** 10.1007/s11832-016-0751-7

**Published:** 2016-06-24

**Authors:** Kate E. Gargan, Catharine S. Bradley, Alexandra Maxwell, Joel Moktar, John H. Wedge, M. Lucas Murnaghan, Simon P. Kelley

**Affiliations:** Division of Orthopaedic Surgery, The Hospital for Sick Children, 555 University Avenue, Toronto, ON M5G 1X8 Canada

**Keywords:** Pediatric orthopedics, Developmental dysplasia of the hip, Pavlik harness, Simulated learning, Parent education

## Abstract

**Background:**

The Pavlik harness is the most common initial treatment for developmental dysplasia of the hip worldwide. During treatment, parents are required to re-apply the harness at home. Teaching parents how to apply the harness is therefore paramount to success. While simulated learning for medical training is commonplace, it has not yet been trialed in teaching parents how to apply a Pavlik harness.

**Methods:**

A group of parents underwent a simulated learning module for Pavlik harness application. Parents were evaluated pre- and post-exposure and at one month after testing. A validated objective structured assessment of technical skill (OSATS) and a global rating scale (GRS) specific to Pavlik harness application were used for evaluation. A control group of parents was also tested at both time points. A clinical expert group was used to determine competency. ANOVA and *t* tests were used to assess differences between groups and over time.

**Results:**

Parent scores on the OSATS improved to the level of expert clinicians both immediately post-intervention and at retention testing. However, on the GRS, only half were considered competent due to their inability to achieve the required hip positions. The control group did not improve nor were they considered competent.

**Conclusions:**

The use of a simulated learning module improves both the confidence and skill level of parents in the application of the Pavlik harness. However, the challenges parents face in understanding the more detailed subtleties of medical care suggest that they still require an appropriate level of supervision by clinicians to ensure effective treatment.

**Electronic supplementary material:**

The online version of this article (doi:10.1007/s11832-016-0751-7) contains supplementary material, which is available to authorized users.

## Introduction

Developmental dysplasia of the hip (DDH) encompasses a spectrum of hip abnormalities, ranging from ligamentous laxity around the acetabulum to irreducible dislocation [1, 2]. The Pavlik harness, first introduced by Arnold Pavlik in 1944 [3], is a widely used first-line treatment for DDH in infants aged <6 months [4, 5] due to its effectiveness, ease of use and low rate of complications [1, 6–9]. A successful outcome using the device is based on correct application and reapplication [10, 11].

At our institution, the treatment protocol begins with Pavlik harness application in the clinic by experienced clinicians. Although the harness is typically used 24 h/day in the early stages of treatment until the hip is stable, we then offer parents the opportunity to briefly remove the harness for bathing as treatment progresses. However, removal of the harness is not commonly advised in a truly dislocated hip during the first 2–3 weeks until improved stability is confirmed by the treating clinician and does not occur at our institution until 5 weeks from stability being noted. Some institutions also plan for progressive weaning of the harness at later stages of treatment. For daily bathing and harness weaning, parents are responsible for removing and reapplying the Pavlik harness at home. Successful reapplication of the harness by the parents is therefore essential to ensure the hips are maintained in a therapeutic position.

While seemingly simple, the application of a Pavlik harness requires many essential steps to ensure a proper fit [6, 7, 11]. Studies have shown that without educating parents on reapplication, they may reapply the harness incorrectly or fail to reapply it completely [6, 7, 12], both of which can have serious consequences on the outcome of treatment [6, 7, 10, 13]. However, teaching this skill in a clinical context can be fraught with anxieties, stemming in part from parental concern about inadvertently hurting or upsetting their child. Simulated learning using a model and instructional media have been proven to be very effective in teaching this type of skill in a low risk and calm environment [14, 15].

Through international expert consensus, 25 essential steps have been identified in a validated objective structured assessment of technical skill (OSATS) for Pavlik harness application [16]. These steps were utilized for the creation of a simulated learning module that includes an infant doll model, an instructional video and a checklist of essential steps. While it has been shown to improve the ability of residents and allied health professionals to apply a Pavlik harness to the level of expert clinicians, it had not yet been tested on parents [14].

The primary aim of this study was to assess the ability to teach parents to correctly apply a Pavlik harness for DDH using a simulated learning module. The primary hypotheses were that (1) parents would demonstrate an improvement in skill competence after exposure to the module, and (2) parents would demonstrate skill retention at one month after exposure to the module.

## Methods

### Procedure

Parents were recruited to this study if they had no reported prior knowledge or experience with a Pavlik harness. Parents were excluded if their child had DDH or if they had other previous exposure to the harness. Parents were asked to attempt to apply a Pavlik harness to the doll model prior to exposure to the learning module. During these attempts, participants were videotaped using a GoPro video camera (GoPro, Inc. San Mateo, CA, USA) attached to their forehead by the GoPro head strap. The infant doll was of comparable size and weight of a newborn child, was able to be undressed and diapered and had moveable joints at the neck, mid-spine, shoulders, elbows, hips and knees.

Of the 14 parents, 10 underwent exposure to the simulated learning module (test group) and four parents did not (control group). Whilst watching the instructional video, parents had access to the infant model, the Pavlik harness and the skill checklist. Parents were allowed as much time as they felt necessary to learn and practice the application of the harness. The test group was then videotaped reapplying the Pavlik harness. The control group was videotaped reapplying the harness 30 min after their first attempt.

After an interval of one month, the 10 participants who underwent the learning module were again videotaped applying a Pavlik harness, in order to assess the retention of their knowledge regarding the application of the harness. During the 1-month interval, the participants had access to the instructional video. The control group was excluded from this retention test.

A third group made up of three staff orthopedic surgeons, five orthopedic clinical fellows and five orthotists who all work regularly in our DDH clinic comprised the clinical expert group. They were also videotaped applying a harness to the doll model and were used as the comparator/gold standard group for Pavlik harness application.

### Outcome measures

**Fig. 1:**
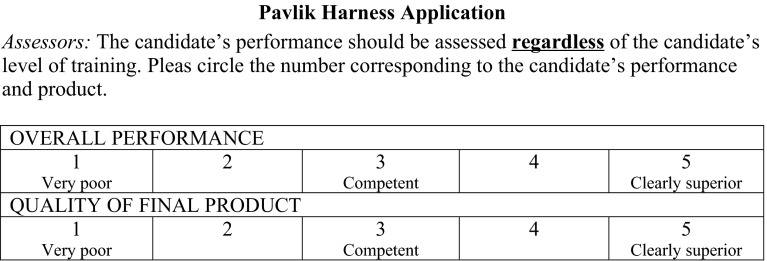
Global rating scales Figure reproduced with permission. Promotional and commercial use of the material in print, digital or mobile device format is prohibited without the permission from the publisher Wolters Kluwer Health. Please contact healthpermissions@wolterkluwer.com for further information [16]

All videos were scored by one content expert (CB) using the validated OSATS specific to Pavlik harness application and a modified global rating scale (GRS) to evaluate both how the skill was performed and the quality of the final product. The OSATS (Electronic Supplementary Material) is a 25-item checklist of skills that was derived from international expert consensus [16]. Items performed correctly are given a score of one for a total possible score of 25. The GRS is scored from very poor (score of 1) to clearly superior (score of 5) (Fig. 1). All three measurements have previously been shown to have excellent reliability and validity [16].

### Statistical analysis

SPSS software ver. 21 (SPSS Inc., Chicago, IL, USA) was used for statistical analysis. One-way ANOVA was used to assess differences between experts, test parents and controls at each available time point. A repeated measure ANOVA was used with the test parents to assess change in scores from baseline, post-intervention and retention testing. A paired sample *t* test was used to assess for change in scores between baseline and follow-up scores for the controls.

## Results

The mean and range of participant scores on the OSATS and GRS throughout the study are presented in Table 1.

**Table 1:** Mean, standard deviation (SD) and range of participant scores on the OSATS and GRS at each available time point

Group		Baseline		Repeated		Retention	
		Mean (SD)	Range	Mean (SD)	Range	Mean (SD)	Range
Parent test	OSATS	6.3 (2.3)	3–11	20 (3.3)	14–25	19.4 (3.0)	15–23
	GRS (performance)	1.1 (0.3)	1–2	2.7 (0.7)	2–4	2.5 (0.7)	2–4
	GRS (product)	1.1 (0.3)	1–2	2.6 (0.7)	2–4	2.1 (0.6)	1–4
Parent control	OSATS	9.7 (2.2)	7–12	9.25 (1.5)	8–11		
	GRS (performance)	1.5 (0.6)	1–2	2 (0.0)	2		
	GRS (product)	1.25 (0.5)	1–2	1 (0.0)	1		
Expert	OSATS	21.2 (2.0)	17–25				
	GRS (performance)	4.4 (0.6)	3–5				
	GRS (product)	4.5 (0.6)	3–5				

### Baseline testing

At baseline testing, there was a significant difference between the expert group scores on all three measurements and both the test parent group (*p* < 0.001) and control parent group (*p* < 0.001). The experts were all considered competent on both the OSATS and GRS, whereas the parents were not.

### Post intervention

Both OSATS and GRS scores for the test parents improved following exposure to the simulated learning module. OSATS scores post-intervention improved to the level of the experts (*p* = 0.291). GRS product and performance scores also improved; however, not to the level of the experts. Only five of the 10 parents after exposure to the learning module were considered to be competent on both GRS scales.

The average amount of time that the test parents took to apply the harness increased post-intervention from 5:04 to 7:29 min. The mean amount of time for the expert group was 5:19 min. A post-intervention survey completed by the parents in the test group revealed that parents felt that their ability to apply a Pavlik harness had improved, with none rating themselves as very weak but none reported being very good either. All, however, reported being more confident about using the Pavlik harness at home.

### Retention testing

On retention testing, the parent OSATS scores remained at the same level as the expert scores; however, at this time point GRS scores were worse with only two of the 10 parents considered competent on both GRS performance and GRS product.

### Control testing

The OSATS and GRS scores for the control group did not significantly change from their initial scores; none were considered competent on either the OSATS or the GRS scales. The time taken by the control group reduced at repeat testing from 4:17 to 3:27 min.

## Discussion

When used appropriately the Pavlik harness is an effective, non-invasive method used to treat DDH [5, 8]. However, in order for Pavlik harness treatment to be effective, parental co-operation, including an understanding of how to correctly apply the harness, is essential [6, 17]. In this study, we described the use and evaluation of a simulated learning module to teach parents Pavlik harness application.

Our results show that the parents improved after exposure to the learning module; however, the majority of parents were still not considered competent on the GRS rating scale. Both the OSATS and the GRS were used because the GRS simply discriminates between better overall performance and quality of product, and gives no specific feedback as to what had been completed accurately and in which areas learners can improve. An analysis into the combined ratings of each participant on the OSATS and the GRS lends significant insight into the challenges parents face in using this device and the support they require to ensure success. Those who were not considered competent on the GRS specifically struggled on items of the OSATS related to achieving the required hip positions.

Despite the success in teaching parents the steps of correct Pavlik harness application using a simulated learning module we also believe it is essential that healthcare providers should still retain a role in parental education, particularly in assisting with setting of the required hip angles. Furthermore, to ensure the hip angles remain correctly set on the Pavlik harness, parents should avoid altering the leg straps on their child’s harness. Instead, they should be advised to only undo the chest strap when removing it. However, as an alternative, markings can also be made on the straps at each visit by the healthcare provider to ensure the harness is repositioned correctly. Accordingly, instructions for marking the straps are included in the teaching video as this is performed in most clinics and is considered very useful for reapplication.

The time taken for the parents in the test group to apply the harness increased after watching the instructional video. The reason for this was because numerous essential steps were initially omitted by the parents, such as measuring the chest to evaluate the correct size of harness, undressing the infant prior to harness application, and/or marking the straps on the harness for reapplication. These steps were learned and performed after exposure to the learning module, and therefore the process took longer, as parents were more thorough in harness application. However, we would expect the clinician to determine the size of the harness at the outset of treatment and to explain to the parent any changes made to the harness throughout treatment. The parents in the control group took less time to apply the harness for the second time. This is likely to have been because the parents were more familiar with the Pavlik harness and so had a clearer idea of how to approach it. These findings demonstrate how exposure to the harness alone can alter the outcome of the application process. The decrease in time needed to apply the Pavlik harness for the control group did not correlate with an improvement in proper application, as there was no increase in scores on either their performance or quality of product.

The feedback from parents regarding the learning module was that some of the language was too complex such as ‘abduction by gravity’ and that the intended position of the hips was difficult to understand and achieve despite the video clearly demonstrating each step of harness application. This was likely because the learning module was initially developed for the education of clinicians. Based on this feedback, we intend to simplify the narration of the instructional video and add graphics, such as arrows to indicate which joints are in question. Even with these adjustments to optimize the learning module for parents, the simulated learning module alone should still be supplemented by healthcare provider support when educating parents in the application of the Pavlik harness. This should include having the clinician check that the parent is capable of reapplying the harness in the correct position if removal is considered appropriate.

## Conclusion

Simulated learning is becoming commonplace and has been shown to be very successful in training medical professionals [14, 15]. In some medical centers there have been recent advances in using simulated learning with parents; however, to our knowledge this is the first study to evaluate the efficacy of this mode of teaching with parents. Our study revealed that the use of a simulated learning module along with an OSATS checklist improves both the confidence and skill level of parents in the application of the Pavlik harness. Future studies will determine the clinical impact of this finding which may include a decrease in Pavlik harness failure in dislocated hips. However, the challenges that parents face in understanding the more detailed subtleties of care suggest that while simulated learning is a key adjunct in the teaching of parents, they still require an appropriate level of supervision by a health care professional to ensure effective treatment.

## Electronic supplementary material

Below is the link to the electronic supplementary material.

Supplementary material 1 (DOCX 23 kb)
