# Supplementary material for: Education of parents in Pavlik harness application for developmental dysplasia of the hip using a validated simulated learning module
Source: J Child Orthop. 2016 Jun 24;10(4):289–93. doi: 10.1007/s11832-016-0751-7 (PMC4940246; doi:10.1007/s11832-016-0751-7)
Supplement: Supplementary file 1 — Supplementary material 1 (DOCX 23 kb) [file 11832_2016_751_MOESM1_ESM.docx]

**OSATS: Application of a Pavlik Harness**

*Instructions to Candidates:*The patient has been diagnosed with DDH.

Apply a Pavlik Harness.

| **ITEM** | **Not done/**  **Incorrect** | **Done/**  **Correct** |
| --- | --- | --- |
| **Application Set up:** | | |
| 1. The baby should be undressed. A diaper/nappy and a single thin layer body garment can remain. | **0** | **1** |
| 1. The correct size harness should be chosen. While not essential, the baby’s chest circumference can be measured at the nipple line using a tape measure as a guide. | **0** | **1** |
| 1. If the baby is on the border of 2 sizes of harnesses, the larger size should be chosen. | **0** | **1** |
| **Halter, Chest and Shoulder Straps:** | | |
| 1. The straps on the harness halter should be opened. | **0** | **1** |
| 1. The opened harness halter should be placed on the bed, front side facing up. | **0** | **1** |
| 1. The baby should be placed supine on top of the halter. | **0** | **1** |
| 1. The chest strap should be brought around the chest and secured at the nipple line. | **0** | **1** |
| 1. The chest strap should be checked by being able to comfortably insert two fingers inside the strap. | **0** | **1** |
| 1. The shoulder straps should be checked to cross posteriorly, then brought over the shoulders and threaded through the buckles on the chest strap. | **0** | **1** |
| 1. The shoulder straps should be secured to keep the chest strap at the level of the nipple line around the entire chest wall. | **0** | **1** |
| **Stirrups, Anterior and Posterior Straps:** | | |
| 1. The foot stirrup straps should be opened. | **0** | **1** |
| 1. The foot stirrups should be applied to the correct foot. | **0** | **1** |
| 1. The foot stirrup straps should be secured around the lower leg. | **0** | **1** |
|  | **Not done/**  **Incorrect** | **Done/**  **Correct** |
| 1. Each foot needs to be held in the foot piece/arch support using the provided sock or a soft shoe. | **0** | **1** |
| 1. The anterior (hip flexion) straps should be pulled through the correct buckle on each side. | **0** | **1** |
| 1. The line of pull of the anterior straps should follow the anterior axillary line on each side. | **0** | **1** |
| 1. The right and left anterior straps should be adjusted and secured with the hips in 90˚ to 110˚ of flexion. | **0** | **1** |
| 1. The posterior (adduction limiting) straps should be pulled through the correct buckle on each side. | **0** | **1** |
| 1. The posterior straps should be adjusted and secured to allow for abduction by gravity (not forced abduction). | **0** | **1** |
| 1. The posterior straps should be adjusted to restrict hip adduction beyond neutral. | **0** | **1** |
| 1. The final position of each hip is re-checked once all straps are secured. | **0** | **1** |
| **For Future Reapplication:** | | |
| 1. The anterior (hip flexion) straps are marked or taped where they have been secured for reapplication. | **0** | **1** |
| 1. The shoulder straps are marked where they have been secured for reapplication. | **0** | **1** |
| 1. The chest strap is marked where it has been secured for reapplication. | **0** | **1** |
| 1. The posterior straps are marked or taped where they have been secured for reapplication. | **0** | **1** |

**MAXIMUM TOTAL SCORE (25)**

**GIVEN SCORE**

Material reproduced with permission. Promotional and commercial use of the material in print, digital or mobile device format is prohibited without the permission from the publisher Wolters Kluwer Health. Please contact healthpermissions@wolterkluwer.com for further information.

Bradley CS, Moktar J, Maxwell A, Wedge JH, Murnaghan ML, Kelley SP. A Reliable and Valid Objective Structured Assessment of Technical Skill for the Application of a Pavlik Harness Based on International Expert Consensus. Journal of Pediatric Orthopaedics. 2015. Epub June, 2015 ahead of print. doi: 10.1097/BPO.0000000000000557
